# Nanotechnology as a New Therapeutic Approach to Prevent the HIV-Infection of Treg Cells

**DOI:** 10.1371/journal.pone.0145760

**Published:** 2016-01-19

**Authors:** Didiana Jaramillo-Ruiz, Francisco Javier De La Mata, Rafael Gómez, Rafael Correa-Rocha, Mª Ángeles Muñoz-Fernández

**Affiliations:** 1 Laboratorio InmunoBiología Molecular, Hospital General Universitario Gregorio Marañón, Instituto de Investigación Sanitaria Gregorio Marañón, Spanish HIV HGM BioBank, Networking Research Center on Bioengineering, Biomaterials and Nanomedicine (CIBER-BBN), Madrid, Spain; 2 Departamento de Química Inorgánica and Química Orgánica, Universidad de Alcalá, Networking Research Center on Bioengineering, Biomaterials and Nanomedicine (CIBER-BBN), Madrid, Spain; University of California, San Francisco, UNITED STATES

## Abstract

**Background:**

HIV-1 has proved to infect regulatory T cells (Treg) modifying their phenotype and impairing their suppressive capacity. As Treg cells are a crucial component in the preservation of the immune homeostasis, we researched that the antiviral capacity of carboxilan dendrimers prevents the HIV-1 infection of Treg and their effects. The phenotype and suppressive capacity of Treg treated or non-treated with carbosilane dendrimers were studied by flow cytometry. Treated and non-treated Treg from healthy donors were infected with HIV-1_NL4.3_. The infection of Treg cells by HIV-1, and protective effect of two dendrimers were determined by measuring antigen p24^gag^ in the supernatant of the culture and intracellular.

**Results:**

The Treg cells were treated with cationic and anionic carbosilane dendrimers. The results showed that both dendrimers did not modify the phenotype and functionality of Treg cells compared with non- treated Treg cells. Anionic dendrimers showed high biocompatibility with normal activity of the Treg cells and in antiviral assays. These dendrimers were highly active against HIV-1 preventing the infection of Treg, and were able to protect the Treg from the Foxp3 downregulation induced by the HIV-1 infection.

**Conclusions:**

This is the first work showing that the *in vitro* use of anionic dendrimers prevent the HIV-1 replication and the infection of expanded Treg cells in culture, which raises the possibility to use Treg cells therapeutically in HIV-1-infected subjects.

## Background

Regulatory T cells (Treg), a specialized subpopulation of CD4+ T cells, are targets for HIV-1 infection [1,2]. These cells constitute a crucial cellular component of a normal immune system and play a pivotal role in establishing and maintaining self-tolerance and immune homeostasis. Thus, Treg cells have an important role in allergy, autoimmune disease, cancer and infectious diseases [3,4].

The immune hyperactivation associated with HIV-1 infection may lead to erosion, depletion and exhaustion of the CD4+ T-cell pool compromising the specific immune responses against HIV-1. Thus, hyperactivation of the immune system is considered a reliable predictor of AIDS progression [5]. The role of Treg cells in HIV-1 infection is critical because of their potential capacity to suppress HIV-1-specific immune responses [6], but also for their implication preventing hyperactivation of immune system and suppressing chronic inflammation [7,8]. We demonstrated that HIV-1 not only directly infects Treg cells, but also deregulates the function and phenotype that define these cells [9,10]. The loss of Treg suppressive function could be responsible for the hyperactivation of immune system associated to the disease, and could be determinant in the progression of AIDS. Therefore, the development of new strategies to prevent the infection of Treg cells by HIV-1, or new approaches to replenish the impaired Treg population could be of great interest to minimize the hyperactivation immune and inflammation, both lead to the generalized degradation of immune system in HIV-infected patients.

Nanoparticles play an increasingly important role in society through a variety of applications ranging from electronics to medicine. Nanocompounds have been widely applied in biomedicine in several ways [11–14]. Dendrimers are multifunctional, hyperbranched and perfectly defined macromolecules [15]. They have shown therapeutic potential in drug delivery, gene therapy, the development of new antiviral agents, etc [11,16]. We have shown that anionic carbosilane dendrimers have antiviral activity *in vitro* and can prevent cell infection [17]. Thus, the antiviral properties of dendrimers could be used therapeutically to prevent the infection of Treg by HIV-1 and to impede the effects of HVI-1 on the phenotype and functionality of these cells [18].

The isolation of Treg cells, *ex vivo* expansion and subsequent reinfusion has proved in animal models to be a successful approach to prevent rejection of transplant and autoimmune disorders. Therefore, the therapy with Treg cells could be an attractive target with potential to control the immune hyperactivation, inflammation and other disorders in HIV-1-infected subjects. However, the fact that Treg cells from subjects can be infected and impaired by the HIV-1 constitutes a strong restriction to the *ex vivo* expansion of functional Treg cells from infected subjects. The employment of dendrimers to impede re-infection of Treg in culture could be of interest in this therapeutic approach. Then, the aim of this research is to determine the antiviral capacity of carbosilan dendrimers to impede the infection of Treg cells by HIV-1 and the effects on their phenotype and suppressive capacity.

## Material and Methods

### Human samples

Blood samples were obtained from buffy coats of healthy donors from the Transfusion Center of Madrid respecting national guidelines [19]. Peripheral blood mononuclear cells (PBMC) were isolated with standard Ficoll gradient (Rafer, Spain) and cultured following the current procedures of the Spanish HIV HGM BioBank [20].

### Dendrimers synthesis

The carbosilane dendrimers were synthesized by the Inorganic Chemistry group of UAH (Madrid, Spain) as previously described and obtained as a white water-soluble solid [21]. The dendrimers were dissolved in phosphate buffered saline (PBS) (Lonza) at the working concentrations just before using. The chemical structures of the first (1G-03NN12) and second (2G-NN16 and 2G-03NN24) generation of cationic carbosilane dendrimers and the second (2G-S24P and 2G-S16) and third (G3-S16) generation of anionic carbosilane dendrimers are shown in S1 Fig.

Cationic dendrimers present in all cases terminal ammonium groups that were bonded to the dendritic skeleton through a Si-O bond that can be slowly hydrolyzed in the presence of water or protic solvents. The differences between them lie on the number of terminal ammonium groups depending on the generation and in the nature of the core that affect to the flexibility of their structure. In the case of 2G-NN16 the core is a silicon atom with four branches emerging from it, whereas for 1G-O3NN12 and 2G-O3NN24 the core is a more rigid polifenolic ring with only three branches emerging from it, that lead to an opener structure.

Regarding to the anionic dendrimers, the three selected dendrimers present sulfonate terminal groups with also some differences between them. 2G-S16 and 3G-S16 are anionic dendrimers with a silicon atom at the core and 16 negatives charges prepared using different synthetic ways. For the 2G-S16 the terminal groups are introduced through Michael type addition reactions, whereas in the 3G-S16 dendrimer the terminal groups were introduced by a click chemistry approach based on alkyne/azide coupling. The third anionic dendrimer, 2G-SP24 differs again in the core that is formed by a poliphenolic ring leading to an opener structure.

### Isolation, expansion and viability of the cultured Treg cells

Treg cells were purified by sorter as previously described [9,10]. Then, they were identified as CD4^+^CD25^+^CD127^low^ and flow cytometry analysis of isolated Treg cells indicates that >85% were Foxp3+. The isolated Treg cells were cultured in 24 well plates for 3 days (1x10^6^ per well) with 1m Lof *OpTImizer TM T-Cell Expansion SFM medium* (Gibco, Carlsbad, CA, US) and 200 μl of *Dynabeads Treg expander* (Invitrogen, Carlsbad, CA, US) supplemented with 500 U/ml of recombinant interleukin 2 (rIL-2).

The viability of Treg cells treated or non-treated with the dendrimers was measured by using the Fixable Viability Dye (eBiosciences, San Diego, California, USA) (S2 Fig). The viability of infected and non-infected Treg cells was determined at 3 and 5 days post-infection. The analyses by using flow cytometry of the Treg phenotypes were performed on living cells.

### Dendrimers and culture of Treg cells

Expanded Treg cells were co-cultured with 2G-NN16, 1G-03NN12, 2G-03NN24 cationic carbosilane dendrimers at 5 μM or 2G-S24P, 2G-S16 and G3-S16 anionic carbosilane dendrimers at 10 μM in RPMI 1640 (Bio Whittaker, Verviers, Belgium) supplemented with 10% fetal bovine serum (FBS) (Lonza) and 500 U/ml of rIL-2. All dendrimers were dissolved in PBS (Lonza) and immediately added to the culture of the Treg cells. Then they were incubated for 48 hours with the dendrimers.

### Analysis of the phenotype and suppressive function of Treg cells

Treg cells were stained for 30 min with anti-CD4 APC, anti-CD25-PECy7, anti-CD38-FITC, anti-CD45RO-ECD and anti-HLA-DR-PECy5 (Beckman Coulter, Barcelona, Spain). Fixation and permeabilization for intracellular staining was done with the *Anti-Human Foxp3 Staining Set* (eBiosciences, San Diego, CA, USA), and cells were stained with anti-Foxp3-PE (eBiosciences) or anti-p24 protein (KC57-FITC; Beckman Coulter). Data acquisition was performed in a Beckman Coulter GALLIOS cytometer. The functionality of the Treg cells was studied measuring the capacity of the Treg to suppress the activation of effector cells. Allogenic PBMC were stimulated with anti-CD3/anti-CD28 coated beads (0.5 beads for 10^5^ cells) (Gibco, Van Allen Way, Carlsbad, California) and incubated for 7 hours. Then these cells were co-cultured with previously expanded and treated Treg cells in a ratio of 1:1 in X-VIVO medium supplemented with 10% of AB human serum. The analysis was performed by using the *Regulatory T–Cell Function Kit* (BD Biosciences, San Jose, CA, US), according to manufacturer´s instructions.

### Virus stock production

CXCR4-tropic virus stock HIV-_NL4.3_ was produced by infection of MT2 cells with NL4.3 virus stock coming from previous transient transfection of pNL4.3 in 293T cells (pNL4.3 plasmid come from NIH AIDS Research Program). Infectious titers of viral stocks were evaluated by limiting dilution on HeLa-CD4-LTR-β-gal (MAGI) cells (NIH AIDS Research Program). These cells contain β-gal gene under the control of HIV-1 long terminal repeat (LTR). Viral titers were expressed as infectious units (IU) per milliliter and infection was done through multiplicity of infection (MOI), where MOI of 1 corresponds to 1 IU per one cell.

### Cell culture and HIV-1 infection

The freshly sorted Treg cells were activated with Dynabeads Human Treg Expander (Invitrogen) following manufacturer’s recommendations and treated for 24 hours with dendrimers. The treated and non-treated Treg cells were then infected with X4-tropic HIV-1_NL4.3_ (MOI of 0.1) for 3 hours. These cells were then extensively washed and cultured in the presence of 500 U/ml of rIL-2. The controls of inhibition of HIV-1 replication were performed with zidovuline (AZT) (Retrovir; GlaxoSmithKline, Brentford, UK).

The HIV-1 entry and infection were confirmed by measuring the concentration of p24^gag^ in the culture supernatant by ELISA (Innotest HIV-1 antigen mAb; Innogenetics, Gent, Belgium) or by the intracellular expression of p24 using the KC57 antibody.

### Statistical analysis

Statistical analysis was performed by using GraphPad Prism software. The treated and non-treated Treg cells were compared in each experiment by using the non-parametric Mann- Whitney test. Dendrimer effects on the phenotype of Treg were compared by using the Wilcoxon paired test. *p-*value less than 0.05 was considered significant.

## Results

### Treg viability assay

**Fig 1:**
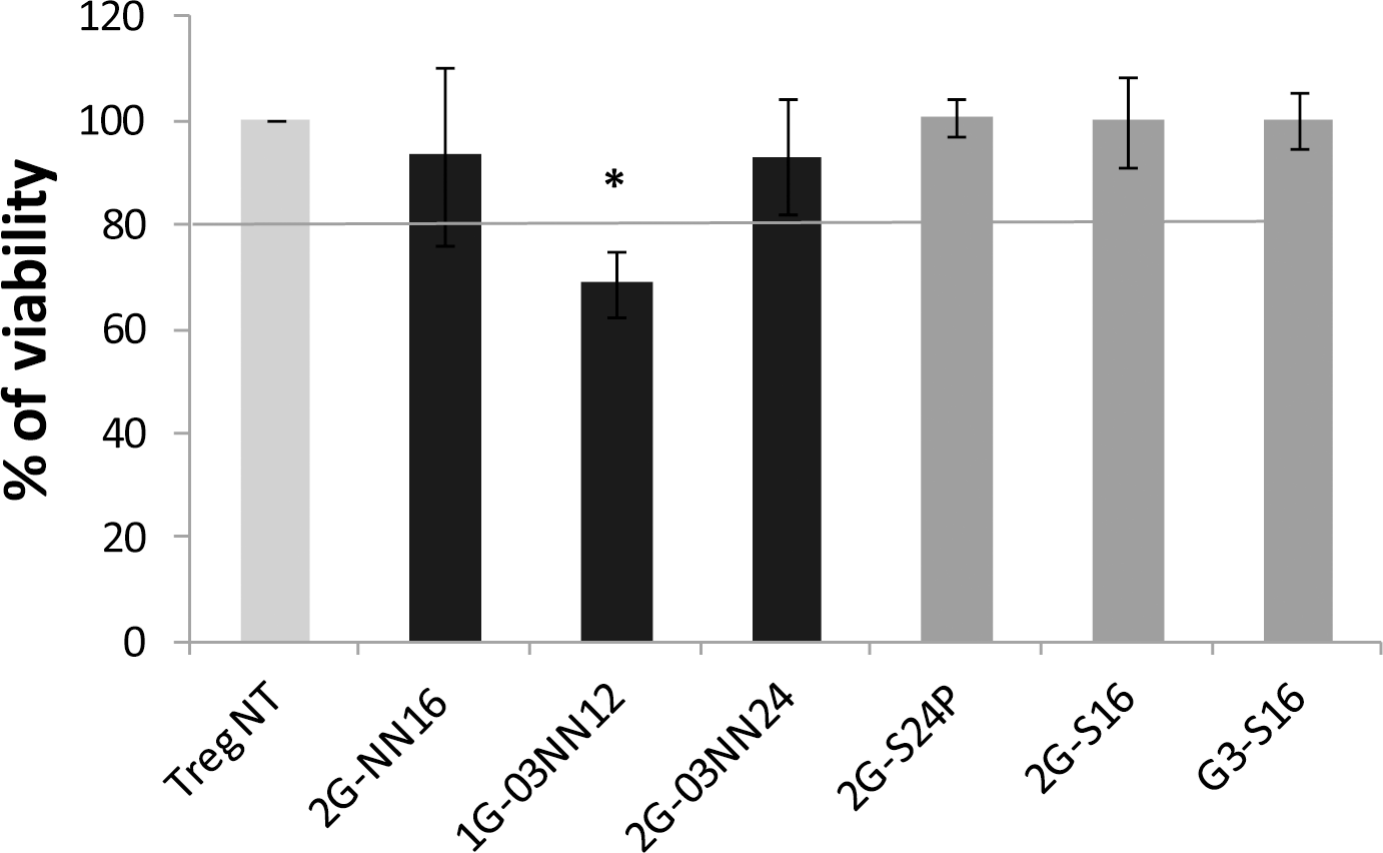
Toxicity of the dendrimers on cultured Treg cells. The percentage of living Treg cells treated with cationic or anionic carbosilane dendrimers was analyzed by flow cytometry after 48 hours of treatment. Viability of Treg were calculated and normalized regarding the non-treated Treg condition. Mean + SEM of 2G-NN16 (n = 8), 1G-03NN12 (n = 5), 2G-03NN12 (n = 7), 2G-S24P(n = 7), 2G-S16 (n = 5), and G3-S16 (n = 4) independent experiments are showed. *p< 0.05 in the Mann Whitney test.

The Treg cells were treated 48 hours with cationic carbosilane dendrimers, which were considered non-toxic at 5 μM, and anionic carbosilane dendrimers, which were considered non-toxic at 10 μM because survival rate were >80% (S2 Fig). The Treg cells treated with 2G-NN16, 2G-03NN24, 2G-S16, 2G-S24P and G3-S16 dendrimers showed a percentage of living cells similar to non-treated Treg cells evaluated by flow cytometry. Only the 1G-03NN12 cationic dendrimer reduced the viability of treated Treg cells below 80%, and was considered toxic at 5 μM (Fig 1, S6A Fig).

### Carbosilane dendrimers have no effect in the Foxp3 expression of treated Treg cells

**Fig 2:**
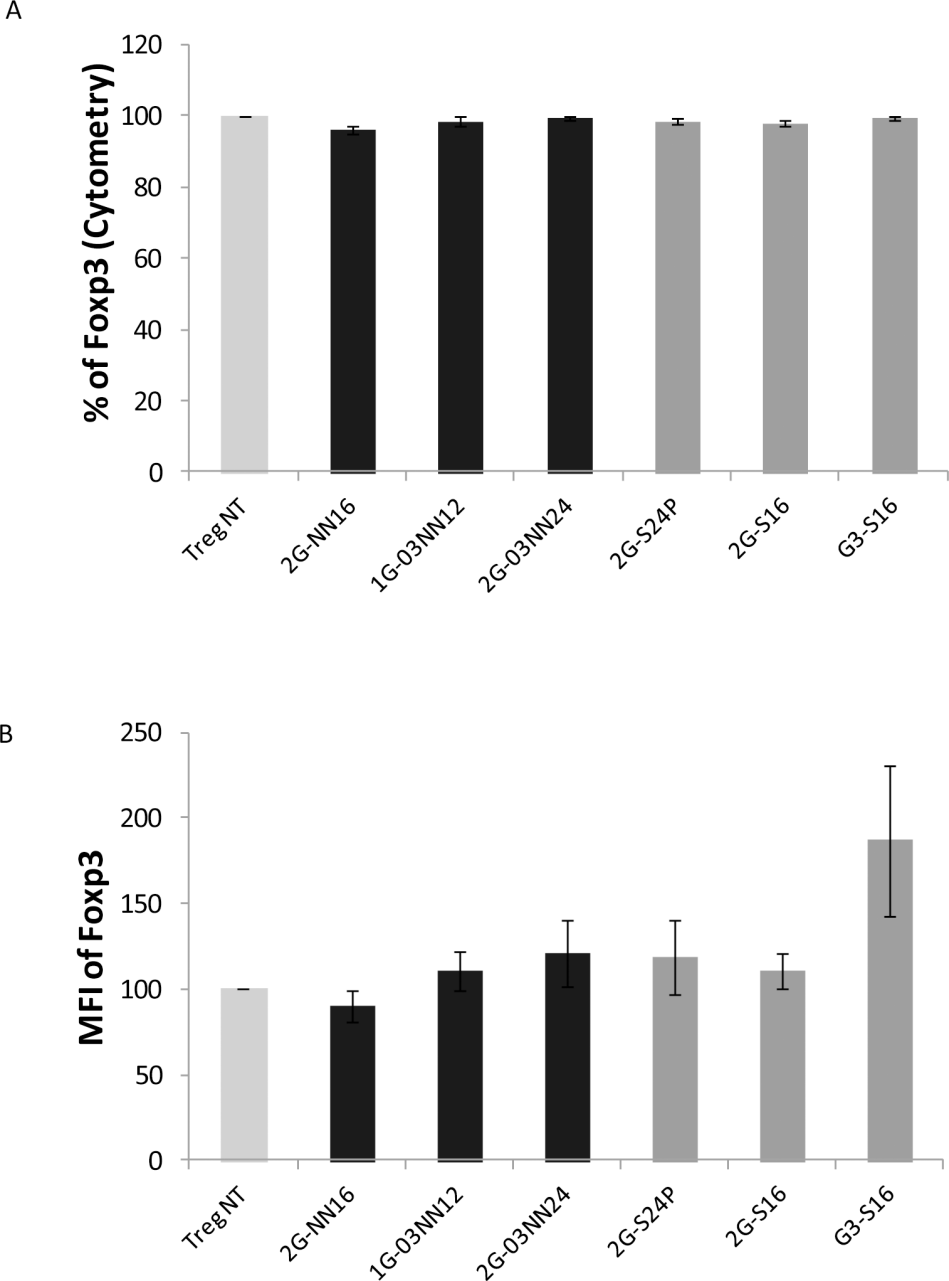
Foxp3 expression on Treg cells was not affected by dendrimers treatment. Percentage (A) and MFI (B) of Foxp3 expression was analyzed in treated Treg after 48 hours of treatment with cationic or anionic dendrimers. Values of Foxp3 expression were calculated and normalized regarding the non-treated Treg (Treg NT) condition that was considered as 100% of expression. Mean + SEM of 2G-NN16 (n = 8), 1G-03NN12 (n = 5), 2G-03NN24 (n = 7), 2G-S24P (n = 7), 2G-S16 (n = 5), and G3-S16 (n = 4) independent experiments are shown. The analyses were performed with the Wilcoxon paired test.

The Treg cells are frequently identified as CD4^+^CD25^+^Foxp3^+^ cells. Foxp3 is the marker that defines Treg cells and plays also a crucial role in the functionality of these cells. Therefore, we studied whether the treatment with cationic or anionic dendrimers for 48 hours modifies the phenotype of the Foxp3 expression in Treg cells. The expression of Foxp3 was not affected by the presence of 2G-NN16, 1G-03NN12 or 2G-03NN24 cationic dendrimers, or 2G-S24P, 2G-S16 and G3-S16 anionic dendrimers at the concentration assayed in the Treg cells compared to the non-treated Treg cells (Fig 2, S3 Fig, S6B Fig). Moreover, the mean fluorescence intensity (MFI) was equally similar in all treated Treg cells compared to the non-treated Treg cells (Fig 2B).

### Analysis of CD4, CD45RO, CD38 and HLA-DR expression in Treg cells treated with carbosilane dendrimers

**Fig 3:**
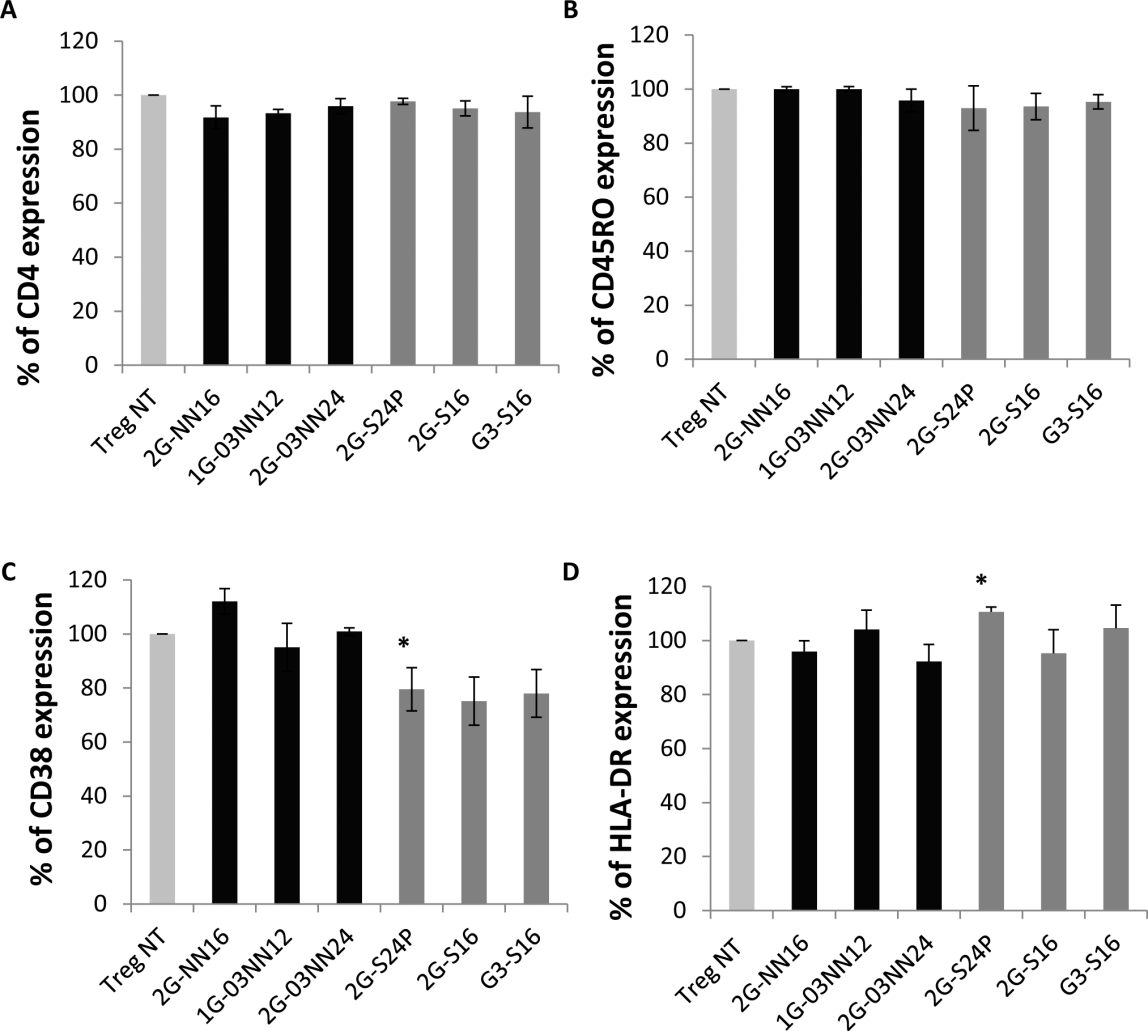
Phenotype expression in Treg cells treated with dendrimers. The percentage of CD4 (A), CD45RO (B), CD38 (C) and HLA-DR (D) expression was analyzed after 48 hours of Treg cells treated with cationic or anionic dendrimers. Values were calculated and normalized regarding the non-treated Treg (Treg NT) condition that was considered as 100% of expression. Average of 2G-NN16 (n = 7), 1G-03NN12 (n = 5), 2G-03NN24 (n = 7), 2G-S24P (n = 7), 2G-S16 (n = 4), and G3-S16 (n = 4) experiment (Mean + SEM). *p<0.05 obtained with the Wilcoxon paired test.

In addition to Foxp3, we analyzed whether the treatment with dendrimers modified the expression of other key markers in Treg cells. The results showed that percentage of CD4 expression was not modified in the Treg treated with 2G-NN16, 1G-03NN12 or 2G-03NN24 cationic dendrimers or treated with 2G-S24P, 2G-S16 or G3-S16 anionic dendrimers (*p>* 0.05) (Fig 3A, S7A Fig).

We also analyzed the expression of CD45RO, CD38 and HLA-DR in Treg cells treated with cationic or anionic dendrimers because these markers are involved in the activation or functional features of Treg cells. The results showed that none of the dendrimers tested modifies the frequency and MFI of CD45RO in treated Treg compared to non-treated Treg cells (Fig 3B; S4A Fig, S7B Fig). The CD38 expression was decreased in Treg treated with 2G-S24P anionic dendrimer (*p*< 0.05), but the rest of dendrimers did not show any effect on CD38 expression (Fig 3C, S8A Fig). Finally, 2G-S24P also produced an increase in the percentage of HLA-DR activation marker (*p<* 0.05), which was not observed with the rest of cationic and anionic dendrimers (Fig 3D, S8B Fig).

### Effect of the carbosilane dendrimers in suppressive capacity of Treg cells

**Fig 4:**
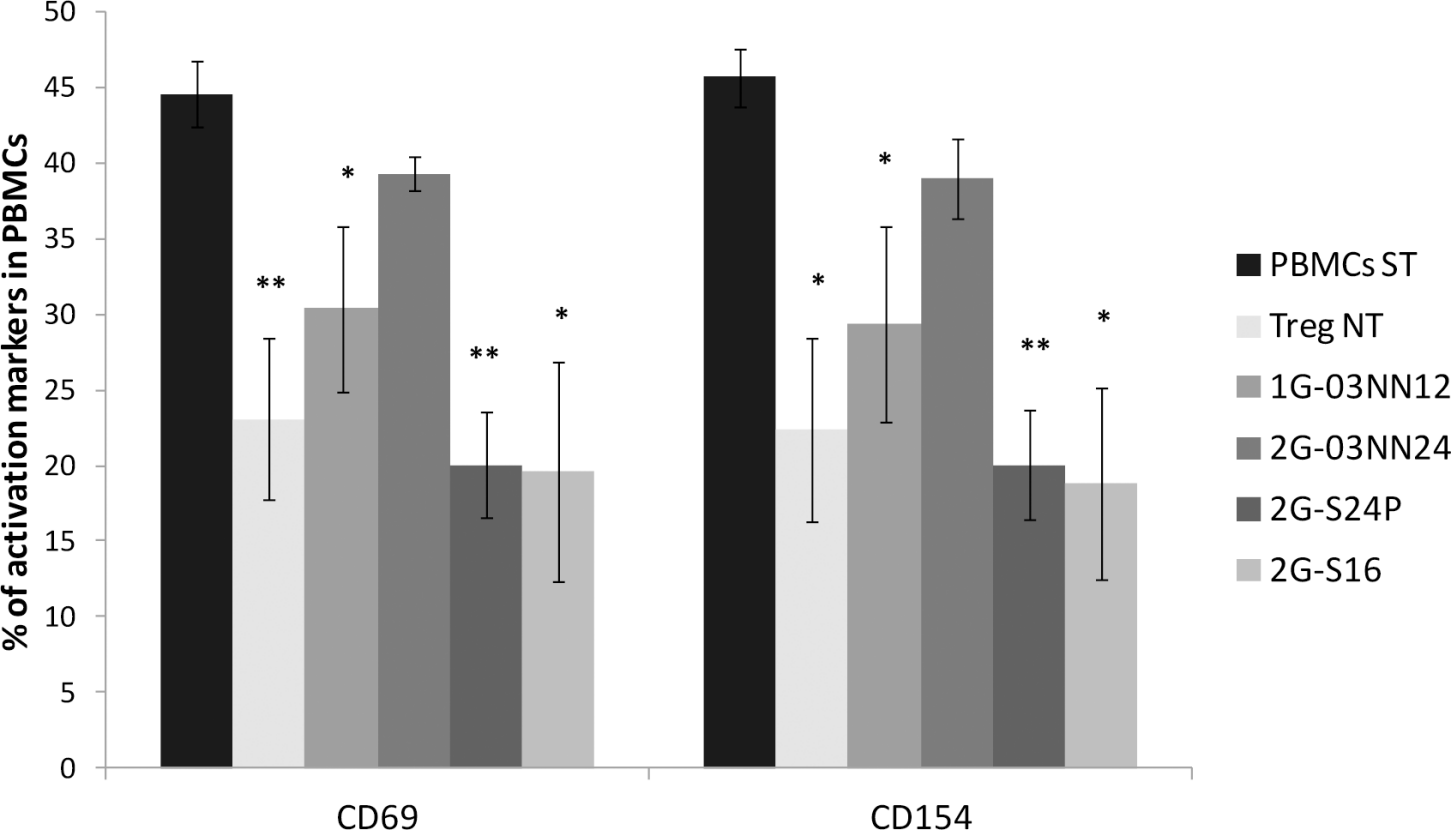
Suppressive capacity of Treg cells treated with dendrimers. Mean + SEM of the percentage of CD69 and CD154 activation markers in stimulated allogeneic PBMC alone (PBM ST) and co-cultured with Treg cells treated or non-treated (Treg NT) with cationic or anionic dendrimers (Treg: PBMC ratio of 1:1). Treg NT (n = 5), 1G-03NN12 (n = 4), 2G-03NN12 (n = 3), 2G-S24P (n = 5) and 2G-S16 (n = 3).**p<0.01; *p<0.05 with the Mann Whitney test.

The suppressive capacity of Treg cells to inhibit the activation of the PBMC was evaluated by measuring the percentage of expression of CD69 and CD154 markers in stimulated allogeneic PBMC co-cultured with Treg cells treated with 1G-03NN12 or 2G-03NN24 cationic dendrimers and 2G-S24P or 2G-S16 anionic dendrimers [22] The non-treated Treg cells showed a high capacity for suppress the expression of the activation markers in stimulated PBMC compared to stimulated PBMC alone (*p*<0.05), showing that our *in vitro* expanded Treg cells preserved their suppressive activity (Fig 4). The stimulated PBMC in presence of the Treg cells treated with 1G-03NN12 also showed a reduction in the percentage of activation markers, these results suggest that the Treg cells preserved their function in presence of this dendrimer. The co-cultures with Treg cells treated with 2G-03NN24 showed an expression of activation markers similar to PBMC alone, indicating a loss of the suppressive capacity in these Treg cells. Interestingly, the suppressive capacity of the Treg cells treated with the anionic dendrimers 2G-S24 and 2G-S16 at 10 μM was preserved or even higher than the capacity of the non-treated Treg cells (Fig 4).

### Dendrimers protect Treg cells against the HIV-1 infection

We researched whether the antiviral effect of some dendrimers reported in primary cells could also have a protective effect on Treg cells against HIV-1 infection. The 2G-S24P and 2G-S16 anionic dendrimers were selected because these dendrimers did not produce alterations in the viability and Foxp3 expression of Treg cells. Besides, a X4-HIV-1_NL4.3_ strain was used to infect the Treg cells because this strain has previously demonstrated a high HIV-1 infection and replication in Treg cells, and a high effect on the Foxp3 expression [10].

**Fig 5:**
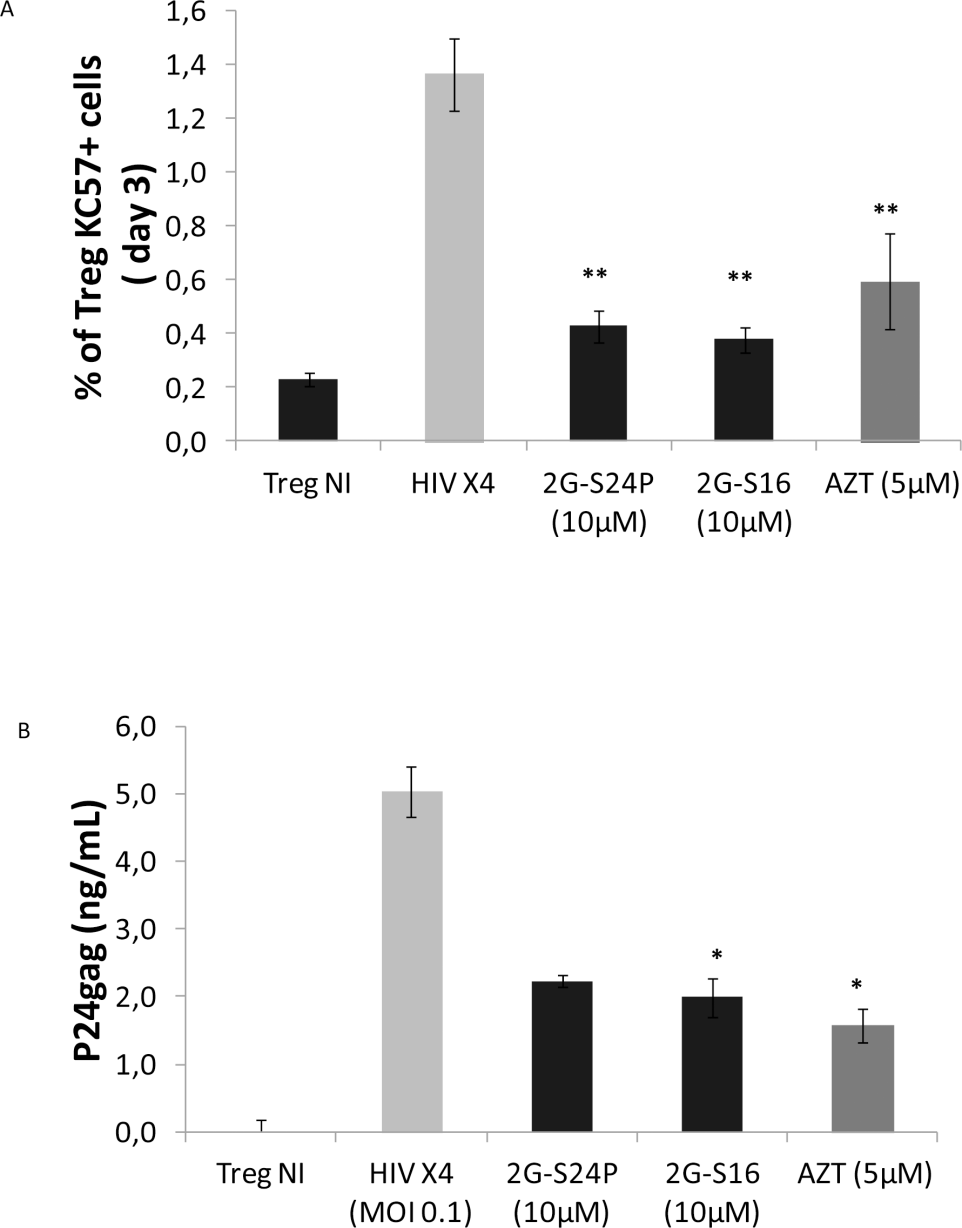
HIV infection of Treg cells treated with dendrimers. (A) Mean ± SEM values of intracellular p24gag at 3 days post-infection determined by frequency of KC57 (p24gag) in Treg infected with X4-tropic HIV_NL4.3_ (MOI 0.1) (n = 6), HIV_NL4-3_ + 2G-S24P (n = 6), HIV_NL4.3_ + 2G-S16 (n = 4) and HIV_NL4.3_ + AZT (n = 4). (B) Mean ± SEM values of p24gag (ng/ml) production at 5 days post-infection determined by ELISA in supernatants of Treg infected with X4-tropic HIV_NL4.3_ (n = 5), HIV_NL4.3_ + 2G-S24P (n = 3), HIV_NL4.3_ + 2G-S16 and HIV_NL4.3_ + AZT (n = 5). **p< 0.01; *p< 0.05 were obtained with the Mann Whitney test.

We confirmed that X4-HIV-1_NL4.3_ strain infected effectively Treg cells by measuring the expression of the viral p24gag protein in the cytoplasm (KC57+ cells) (Fig 5A, S5 Fig). However, this flow-cytometric method has a low sensitivity, because it measures HIV particles replicating into the cells during the short staining period, and do not measure all the particles that has been liberated previously to the supernatant by infected Treg cells, underestimating the frequency of infected Treg. For that we confirmed the infection of Treg cells by detecting a high expression of the viral p24gag protein in the supernatant (Fig 5B) of infected Treg cells. The treatment of the Treg cells with the 2G-S24P and 2G-S16 anionic dendrimers at 10 μM reduced markedly the X4-HIV-11_NL4.3_ infection (*p*<0.01) compared to the non-treated Treg, as reflected by the significant lower values of p24 detected. The inhibition of X4-HIV-1_NL4.3_ infection in 2G-S24P and 2G-S16 treated Treg cells was comparable to the inhibition of the infection obtained in AZT treated Treg cells (Fig 5).

**Fig 6:**
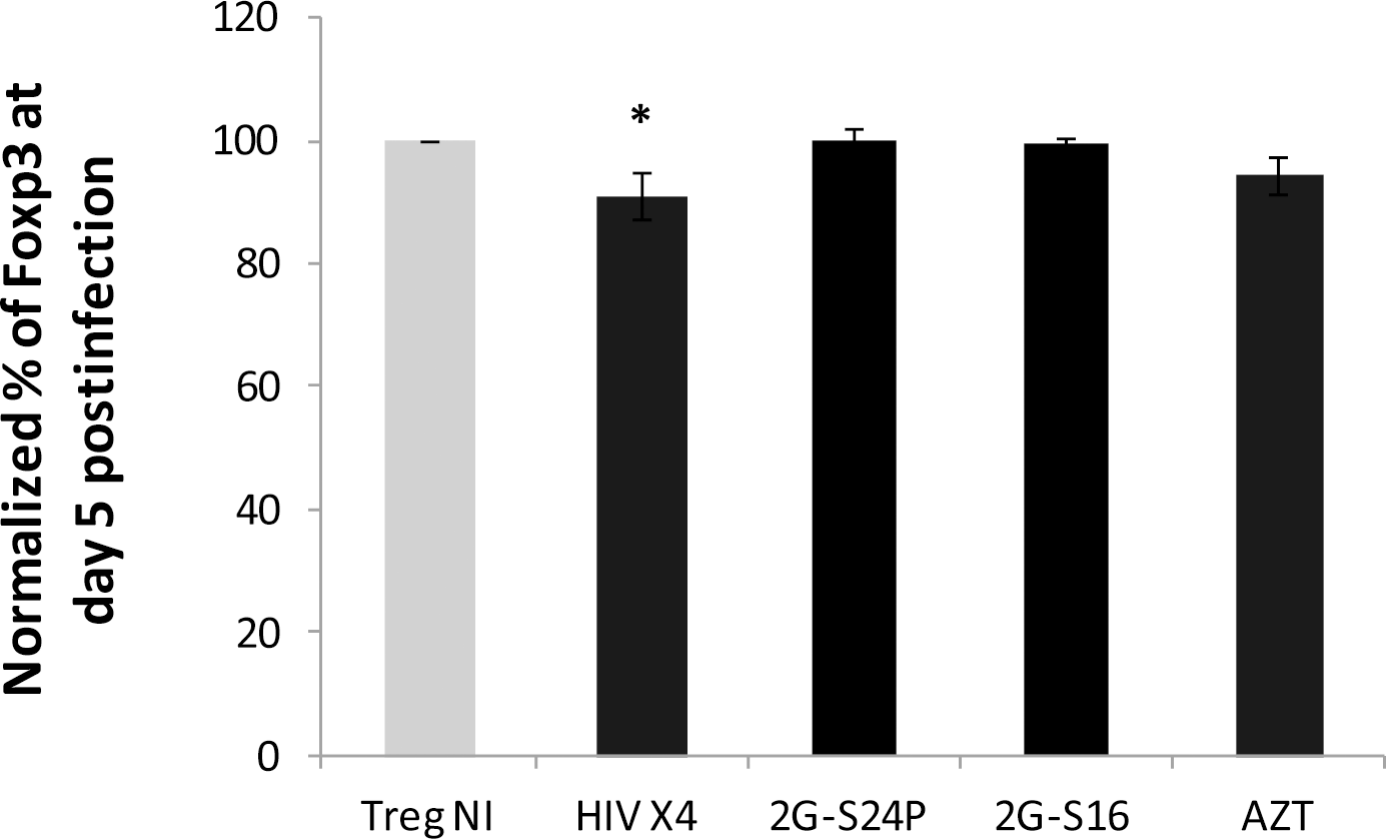
Foxp3 expression in Treg cells infected with HIV-1. Mean ± SEM values of frequency of Foxp3 in Treg infected with X4-tropic HIV_NL4.3_ (MOI 0.1), HIV_NL4.3_ + 2G-S24P (10μM), HIV_NL4.3_ + 2G-S16 (10μM) or HIV_NL4.3_ + AZT (5μM) at 5 days post-infection. Average of Treg NI and X4-tropic HIV_NL4.3_ (n = 6), 2G-S24P (n = 6), 2G-S16 and AZT (n = 3) independent experiments.*p<0.05 obtained with the Mann Whitney test.

Regarding the expression of Foxp3 in Treg cells, our results confirmed that the level of Foxp3 expression was significantly lower in *in vitro* X4-HIV-1_NL4.3_ infected Treg cells (Fig 6). The downregulation of Foxp3 in X4-HIV-1_NL4.3_ infected Treg cells was detected at day 3 (*data not shown*) and day 5 post-infection. However, when Treg cells were pre-treated with the 2G-S24P and 2G-S16 anionic dendrimers at 10 μM, the percentage of the expression Foxp3 level was similar to non-infected Treg after 5 days of X4-HIV-1_NL4.3_ infection (Fig 6). Summing up, the 2G-S24P and 2G-S16 anionic dendrimers protected the Treg cells from the Foxp3 downregulation induced by the HIV-1 infection.

In summary, the treatment of Treg cells with cationic and anionic carbosilane dendrimers did not affect the viability and functionality of the Treg cells, and did not produce important alterations in the phenotype expression of these cells. Therefore, the use of dendrimers as vectors or as inhibitors in Treg cells would be possible without modifying the main characteristics of this relevant population. Moreover, the treatment with anionic dendrimers has demonstrated to be effective in inhibiting the HIV-1 infection of Treg cells, and diminishing the effects on Foxp3 expression produced by the HIV-1 infection.

## Discussion

The role of Treg cells in the HIV-1 infection remains controversial. Treg cells could either suppress HIV-1-specific immunity and thereby promote HIV-1 spread; or alternatively could suppress deleterious HIV-1-associated immune activation decreasing HIV-1 disease progression and preventing the hyperactivation of the immune system, which is considered a reliable predictor of AIDS progression [5]. Recent findings demonstrate that absolute counts of Treg cells are markedly diminished in HIV-1-infected subjects [23–25], and this deficiency is correlated with a generalized immune activation [7], supporting the idea that Treg cells are crucial to maintain the correct homeostasis of the immune system and thus, a deficiency of these Treg cells could contribute to the progression of the HIV-1 infection. In addition, HIV-1-specific Treg cells have been identified in infected patients [26], supporting the relevance of this subset in HIV infection.

We have previously demonstrated that the depletion of Treg cells seems to be mediated by the direct HIV-1-infection of the Treg cells and the consequent disturbance of their phenotype expression and function [9,18], which has been also confirmed by other authors [27]. Therefore, new therapeutic approaches directed to impeding the Treg HIV-1 infection and to preserving this population of cells could be of great interest in the HIV-1-infected subjects. Here, we researched the use of carbosilane dendrimers to protect the Treg cells from the HIV-1 infection and their effects on the phenotype expression of these cells. Since the treatment of Treg cells with these carbosilane dendrimers has not been previously studied, we firstly analyzed whether these dendrimers can modify *per se* the viability and/or phenotype of this cell population.

Due to the peripheral positive charges, cationic carbosilan dendrimers can be useful as carriers for the delivery of nucleic acids or peptides to the cells [11,12,16]. The results showed that 2G-NN16 and 2G-03NN24 cationic dendrimers did not modify the viability of Treg cells, as reported in other primary cells treated with the same dendrimers [16,17]. Only the 1G-03NN12 dendrimer decreased the viability below 80%, which could be explained by the fact that the positive charges can produce destabilization of the cellular membrane, inducing the lysis of the cells [28]. None of the cationic dendrimers tested modified the expression of the markers analyzed, notably the Foxp3 expression, and the treated Treg cells showed a comparable phenotype to the non-treated Treg cells. In addition, we did not observe significant differences in the suppressive capacity of Treg cells treated with 1G-03NN12, but 2G-03NN24 seemed to produce an impairment in the function of Treg cells. At the light of these results we can conclude that, the use of cationic carbosilan dendrimers as carriers in Treg cells is possible because they do not produce marked effects on the phenotype of these cells.

We also researched the effects on Treg cells of a treatment with anionic carbosilan dendrimers. None of the three anionic dendrimers tested decreased the viability of Treg cells, and the treatment with these dendrimers did not modify the percentage of Foxp3 expression. The treatment with the 2G-S24P anionic dendrimer produced slight changes in the frequency of the activation markers CD38 and HLA-DR. The CD38 is considered an activation marker in T cells, but several studies have demonstrated that Treg cells with a high expression of CD38 possess a higher suppressive capacity and survival [29]. On the other hand, this 2G-S24P anionic dendrimer produced an increase in the HLA-DR expression. HLA-DR is also an activation marker and its expression in Treg cells has been related to a higher suppressive capacity [30]. However, the changes observed in these markers in the Treg cells treated with the 2G-S24P dendrimer did not affect the functionality of these cells, which was comparable to non-treated Treg cells, and even produced a slight increase in the suppressive capacity, which in the case of 2G-S24P anionic dendrimer could be a consequence of the increase in the HLA-DR expression of the treated Treg cells. These results indicate that Treg cells can be treated with anionic dendrimers without a substantial effect on the viability, phenotype or functionality of the cells.

Some anionic dendrimers have proved to have antiviral properties. Therefore, we analyzed whether a pre-treatment with anionic dendrimers could reduce the *in vitro* HIV-1 infection of Treg cells, and also prevent the effects of the HIV-1 infection in these cells [10]. For that, we selected the 2G-S16 and 2G-S24P anionic dendrimers because they did not produce remarkable effects on phenotype and functionality of the Treg cells, and these dendrimers have previously proved to prevent the HIV-1 infection in other primary cells [13,17,31]. We confirmed that Treg cells are susceptible of being infected with X4-HIV-1_NL4.3_ strain, as previously described [10]. The pre-treatment with anionic dendrimers prevented significantly the X4-HIV-1_NL4.3_ infection of the Treg cells, and this effect was comparable to the reduction of infection observed by using an AZT treatment. These results are also supported by the reduction in the liberation of viral particles to the supernatant from Treg cells treated with the anionic dendrimers, representing a decreased replication of the X4-HIV-1_NL4.3_ in treated Treg cells. Therefore, the treatment with anionic dendrimers clearly impedes the HIV-1 infection of the Treg cells, and this effect is more marked in the Treg cells treated with the 2G-S16 anionic dendrimer. Studies using computational modeling demonstrated that the 2G-S16 dendrimer could produce stable complexes with the envelope viral glycoprotein gp-120. The entry of the HIV-1 in the target cells is possible by the interaction of the viral gp-120 and the cellular receptor CD4. Therefore, the antiviral capacity of this dendrimer on Treg cells is probably associated to the blockade, at least partially, of the interaction between these two molecules, which is crucial for the HIV-1 entry [17,32]. Moreover, 2G-S16 has also shown the ability to join CXCR4 and CCR5 in the target cell [17], which are co-receptors implicated in the viral entry too. One factor that differentiates the 2G-S16 and 2G-S24P anionic dendrimers is that 2G-S16 has a chemical structure more flexible, which could favor the interaction of the dendrimer with the viral or cellular proteins [17,21]. Additionally, 2G-S24P increased the activation of the Treg cells (higher frequency of HLA-DR), and thus the higher levels of p24 in the Treg cells treated with this dendrimer could be due to a higher replication of HIV-1 associated to the increased activation, and not due to the fact that this dendrimer has a lower antiviral effect.

One of the main effects of HIV-1 infection on the Treg cells is the downregulation of the Foxp3 expression [10], which is crucial for the functionality of the cells. In agreement with the published results, Treg cells infected with X4-HIV-1_NL4.3_ had a lower expression of Foxp3 than non-infected Treg, but the treatment with 2G-S16 or 2G-S24P impeded this effect. The Foxp3 expression in X4-HIV-1_NL4.3_ infected Treg treated with dendrimers was comparable to the expression in non-infected Treg, and the protective effect of these dendrimers was similar to that obtained with the AZT. Therefore, the antiviral effect of the anionic dendrimers “*per se*” protected the Treg cells from HIV-1 infection and prevented the effects of the infection in the phenotype of these cells. Due to the limited number of purified Treg cells, the protective effect of dendrimers in the suppressive capacity was not determined. However, taking in consideration that dendrimers reduce markedly the HIV-1 infection, and prevent the reduction of the Foxp3 expression, directly implicated in the functionality of Treg cells, it is expected that this treatment can also prevent the reduction in the suppressive capacity observed in HIV-1 infected Treg cells.

Modulation of Treg cell function and adoptive Treg transfer are being explored as therapeutic modalities in the context of autoimmune diseases, transplantation and cancer. One of the applications, still highly experimental, of enhancing Treg cells activity *in vivo* would be the transfer of autologous Treg cells, which in numerous animal studies have demonstrated to induce allograft control in transplantation [33]. In addition, adoptive transfer of activated Treg cells provided neuroprotection in an HIV-1 encephalitis mouse model [34]. This therapeutic approach would have a clinical indication outside of the HIV-1 disease (transplantation, autoimmune disease), but also could be crucial to restore the immune homeostasis in HIV-1-infected subjects. However, the capacity of the HIV-1 to infect these cells impairing its functionality restricts the potential therapeutic use of this strategy in HIV-1-infected subjects. In fact, Angin M *et al* describes that Treg cells can be isolated from HIV-1-infected subjects and expanded *in vitro* [35,36], but the capacity of HIV-1 to replicate in these cultured Treg cells compromises the efficacy of these cells and subsequent transfer to the subjects. Here, we clearly demonstrated that the use *in vitro* of anionic dendrimers could prevent the HIV-1 replication and the infection of expanded Treg cells in culture, which theoretically raises the possibility to use Treg cells therapeutically in HIV-1-infected subjects.

In summary, we have demonstrated the antiviral capacity of carbosilan dendrimers to prevent the HIV-1 infection of Treg cells and the effects on their phenotype and suppressive capacity. Our results highlight the use of cationic carbosilan dendrimers as carriers in Treg cells because they do not cause marked effects on the phenotype of these cells. In addition, the use of anionic dendrimers to prevent the HIV-1 replication and the infection of cultured Treg cells raises the possibility to use autologous Treg cells therapeutically in HIV-1 infected subjects.

## Supporting Information

S1 Fig Representation of carbosilane dendrimer structure. (A) Cationic dendrimers: 2G-NN16, 1G-03NN12 and 2G-03NN24. (B) Anionic dendrimers: 2G-S16, 2G-S24P and G3-S16.
(TIF)

S2 Fig Viability in Treg cells treated with carbosilane dendrimers. Histograms showing the viability of non-treated Treg cells compared to Treg cells treated with 2G-03NN12, 2G-03NN24, 2G-S24P and 2G-S16.
(TIF)

S3 Fig Foxp3 expression on treated Treg cells. Dot plots of a representative experiment showing the percentage of Foxp3 in Treg cells treated for 48 hours with carbosilane dendrimers compared to non-treated Treg cells. Numbers represent percentage of positive cells for CD25 and Foxp3 expression.
(TIF)

S4 Fig Phenotype expression in Treg cells treated with dendrimers. The percentage of MFI CD45RO (A), MFI CD38 (B) and MFI HLA-DR (C) expression was analyzed after 48 hours of Treg cells treated with cationic or anionic dendrimers. Values were calculated and normalized regarding the non-treated Treg (Treg NT) condition that was considered as 100% of expression.
(TIF)

S5 Fig Treg cells treated with carbosilane dendrimers are protected from HIV infection. Dot plots are representative experiment showing the percentage of Foxp3+KC57+ cell in Treg cell treated with dendrimers compared to infected HIV Treg cells.
(TIF)

S6 Fig Viability and Foxp3 expression in Treg cells treated with carbosilane dendrimers. (TIF)

S7 Fig CD4 and CD45RO expression in Treg cells treated with carbosilane dendrimers. (TIF)

S8 Fig CD38 and HLA-DR expression in Treg treated with dendrimers. (TIF)
