# Supplementary figures and images for: Nanotechnology as a New Therapeutic Approach to Prevent the HIV-Infection of Treg Cells
Source: PLoS One. 2016 Jan 19;11(1):e0145760. doi: 10.1371/journal.pone.0145760 (PMC4718685; doi:10.1371/journal.pone.0145760)

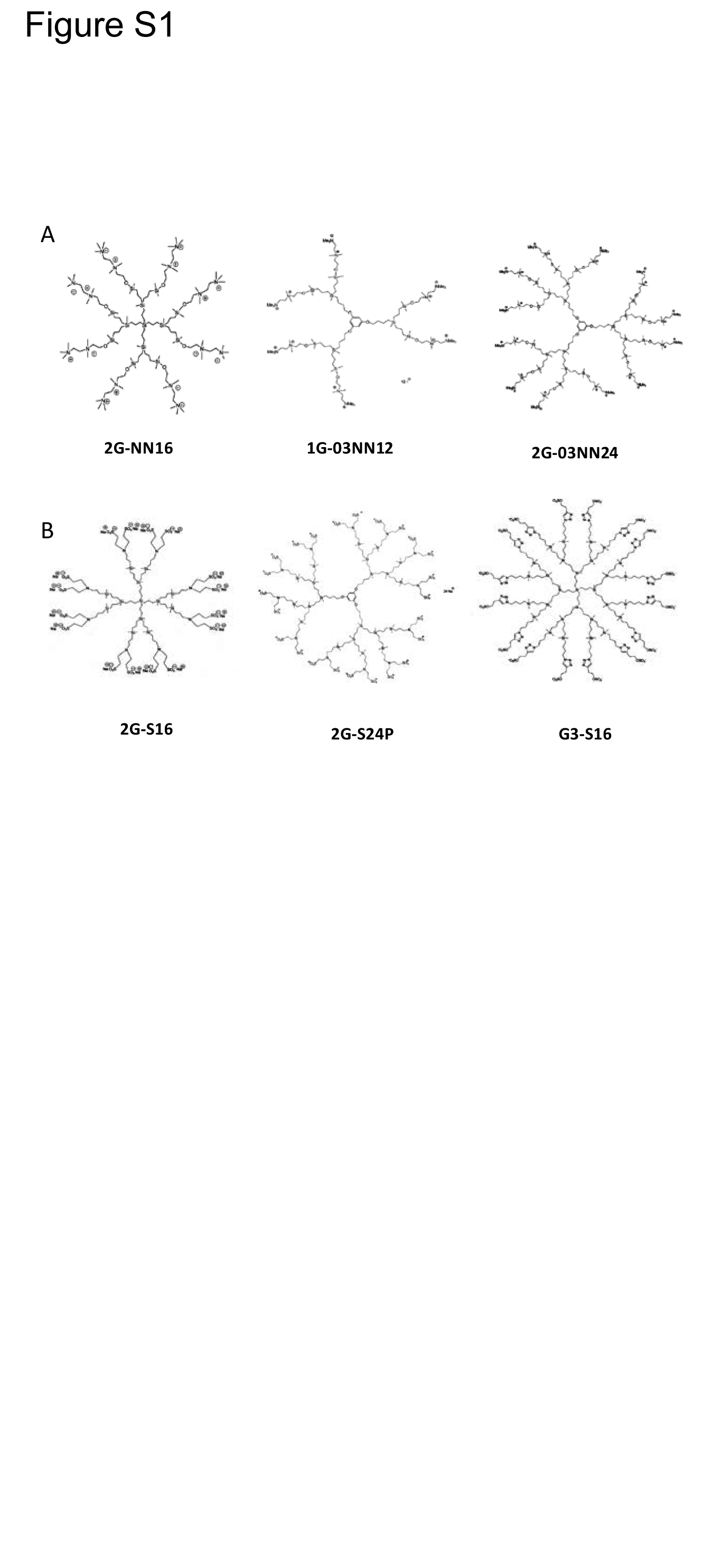

Supplement: S1 Fig — (A) Cationic dendrimers: 2G-NN16, 1G-03NN12 and 2G-03NN24. (B) Anionic dendrimers: 2G-S16, 2G-S24P and G3-S16. (TIF) [file pone.0145760.s001.tif]

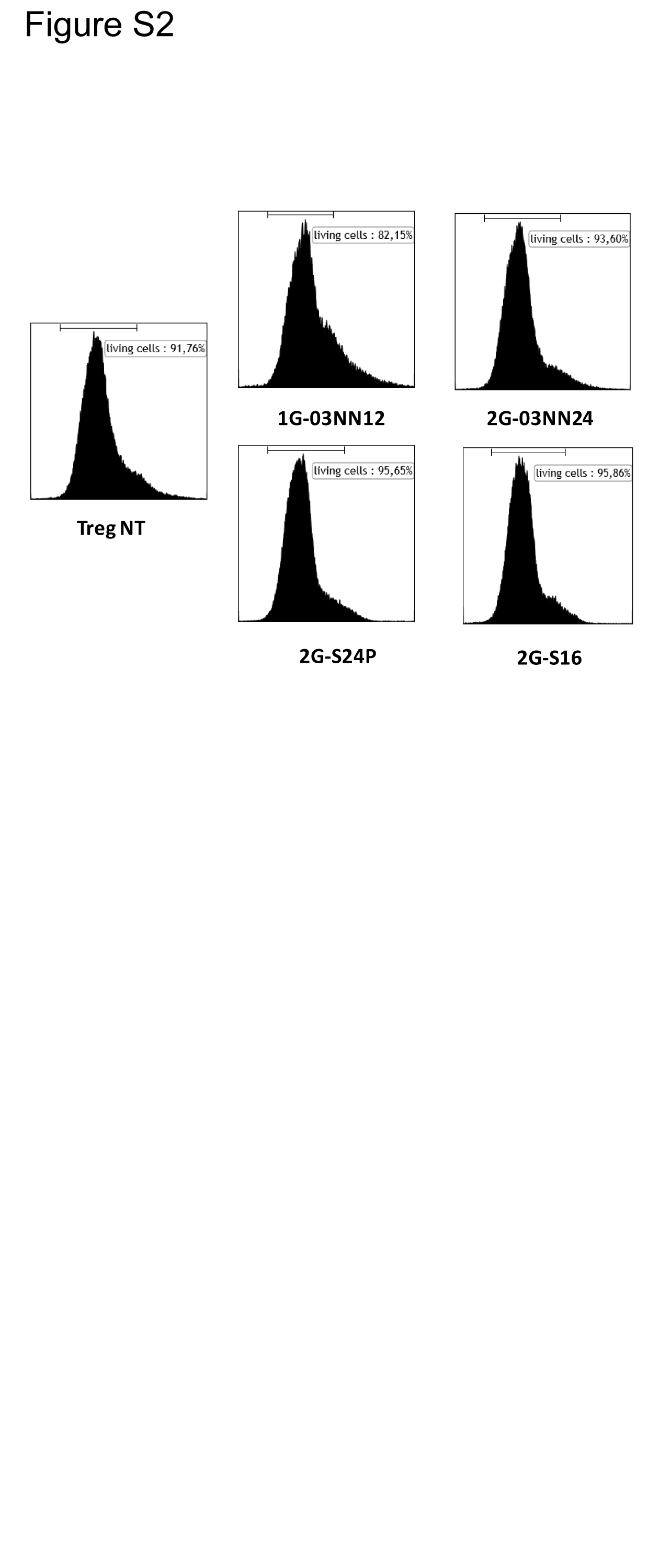

Supplement: S2 Fig — Histograms showing the viability of non-treated Treg cells compared to Treg cells treated with 2G-03NN12, 2G-03NN24, 2G-S24P and 2G-S16. (TIF) [file pone.0145760.s002.tif]

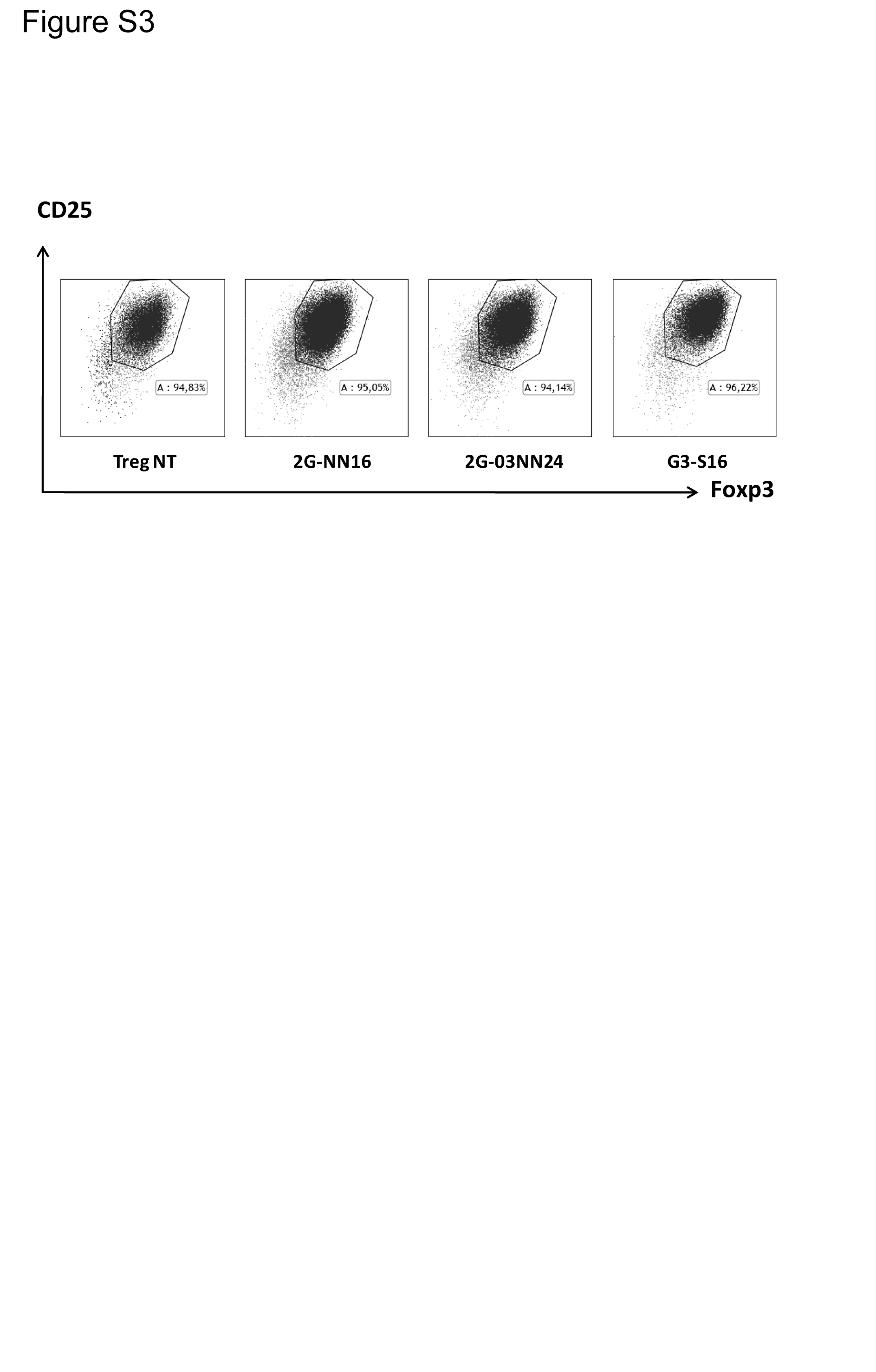

Supplement: S3 Fig — Dot plots of a representative experiment showing the percentage of Foxp3 in Treg cells treated for 48 hours with carbosilane dendrimers compared to non-treated Treg cells. Numbers represent percentage of positive cells for CD25 and Foxp3 expression. (TIF) [file pone.0145760.s003.tif]

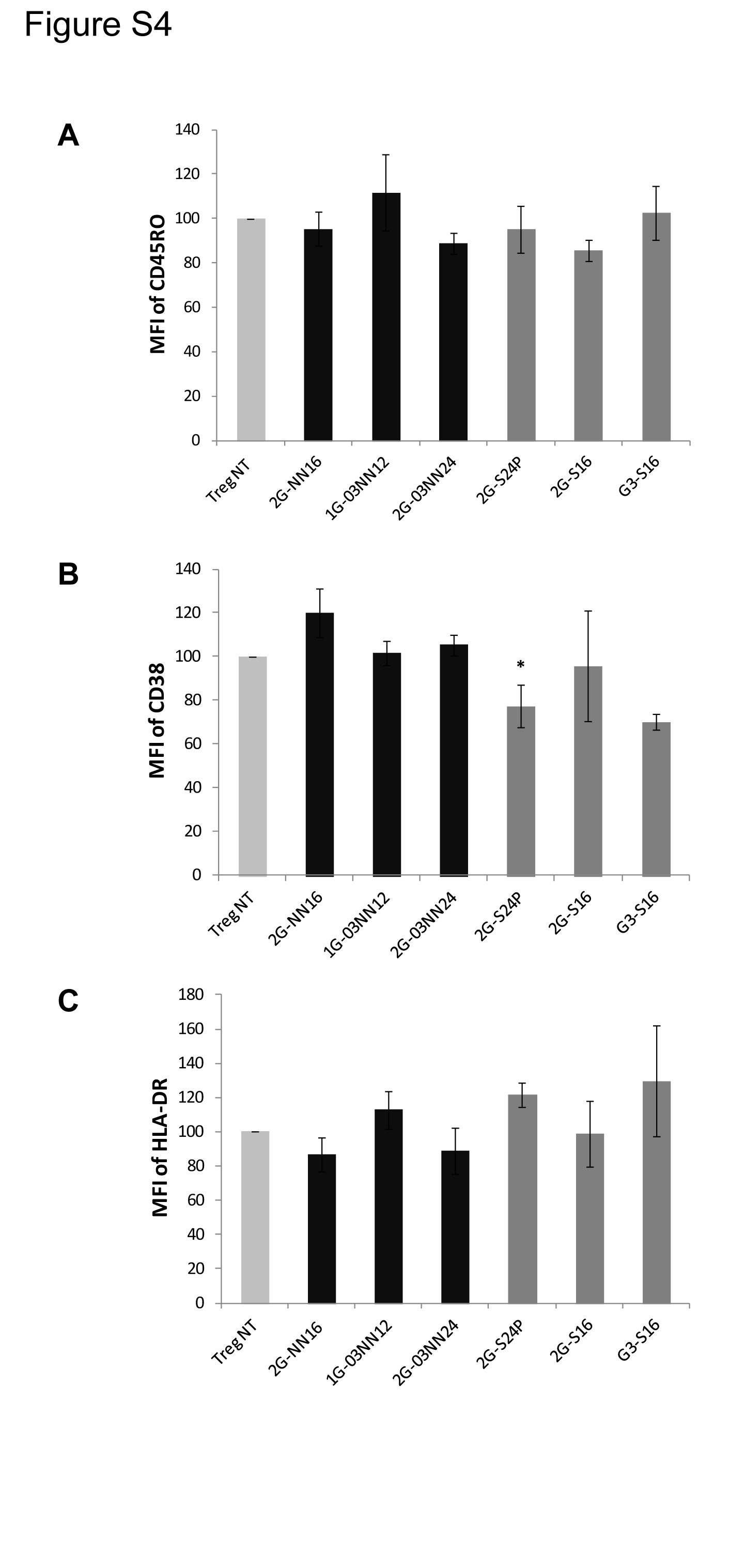

Supplement: S4 Fig — The percentage of MFI CD45RO (A), MFI CD38 (B) and MFI HLA-DR (C) expression was analyzed after 48 hours of Treg cells treated with cationic or anionic dendrimers. Values were calculated and normalized regarding the non-treated Treg (Treg NT) condition that was considered as 100% of expression. (TIF) [file pone.0145760.s004.tif]

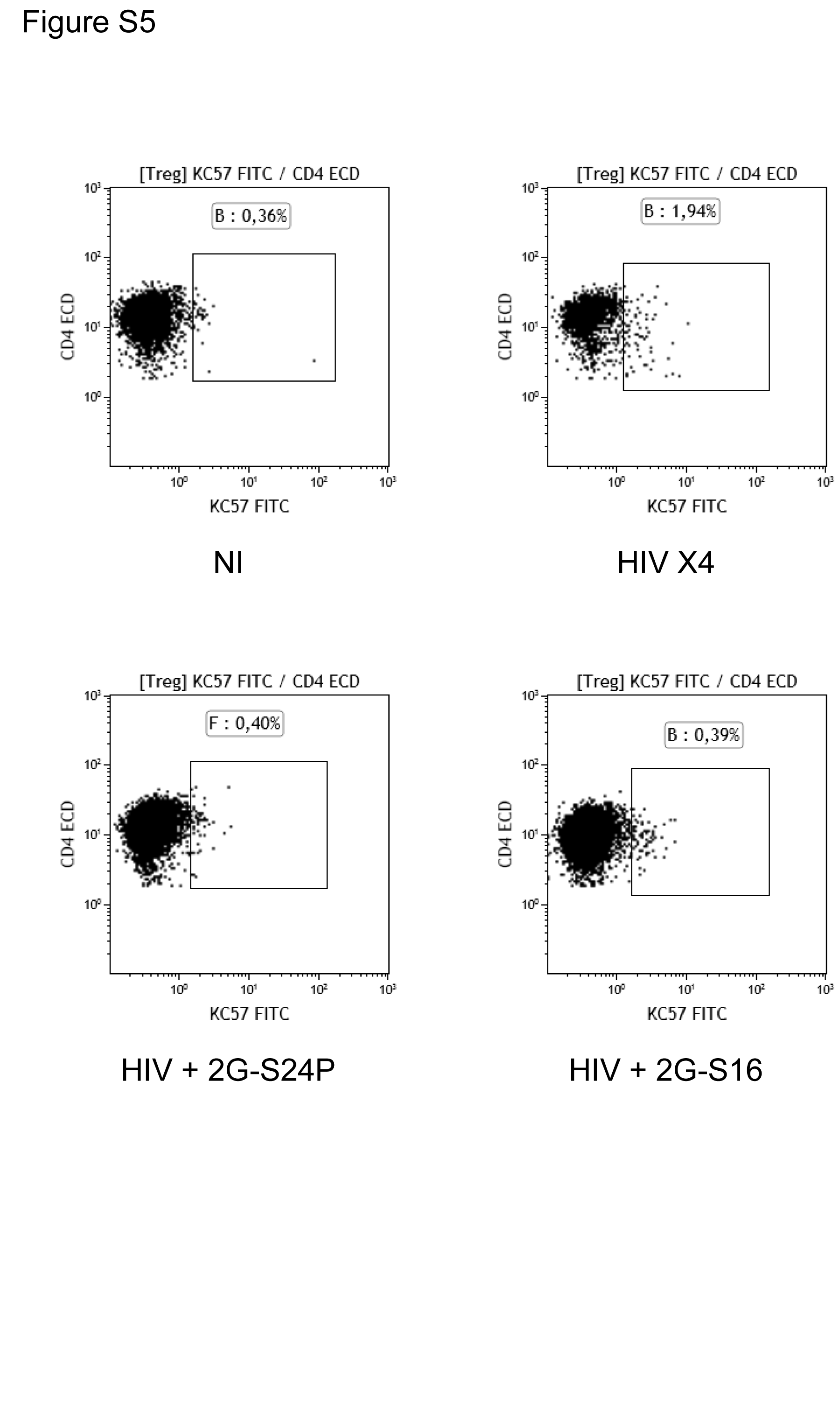

Supplement: S5 Fig — Dot plots are representative experiment showing the percentage of Foxp3+KC57+ cell in Treg cell treated with dendrimers compared to infected HIV Treg cells. (TIF) [file pone.0145760.s005.tif]

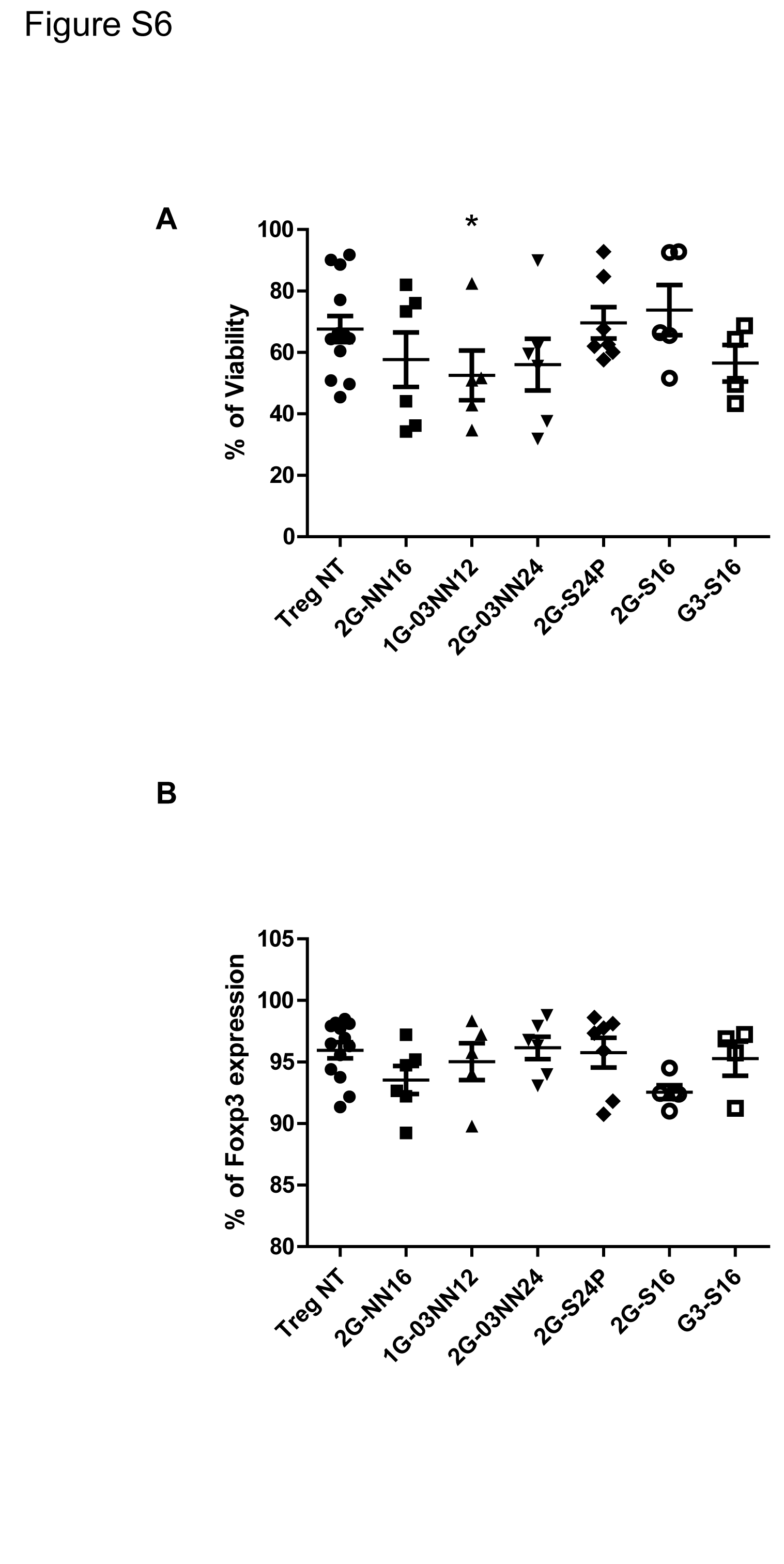

Supplement: S6 Fig — (TIF) [file pone.0145760.s006.tif]

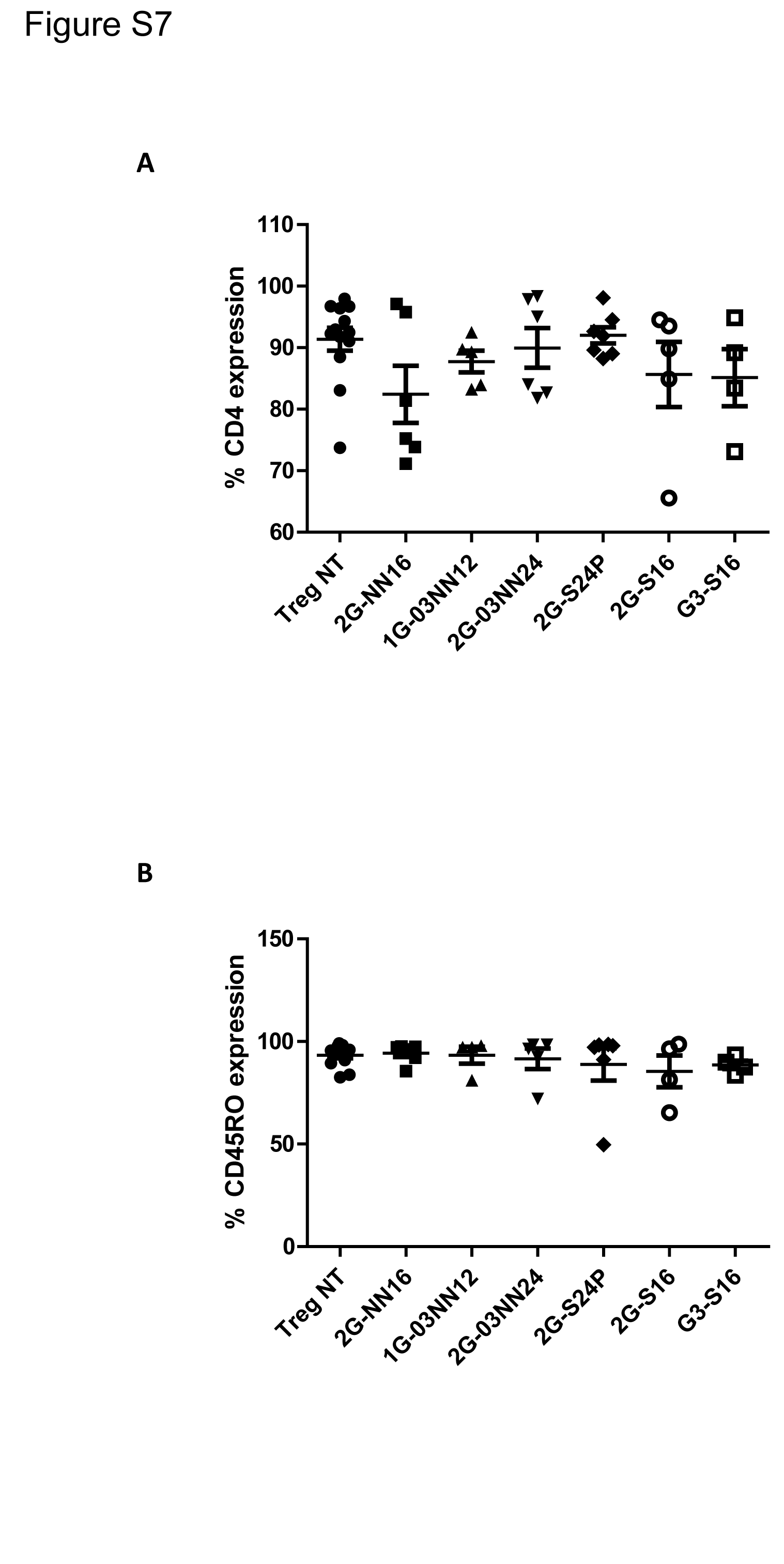

Supplement: S7 Fig — (TIF) [file pone.0145760.s007.tif]

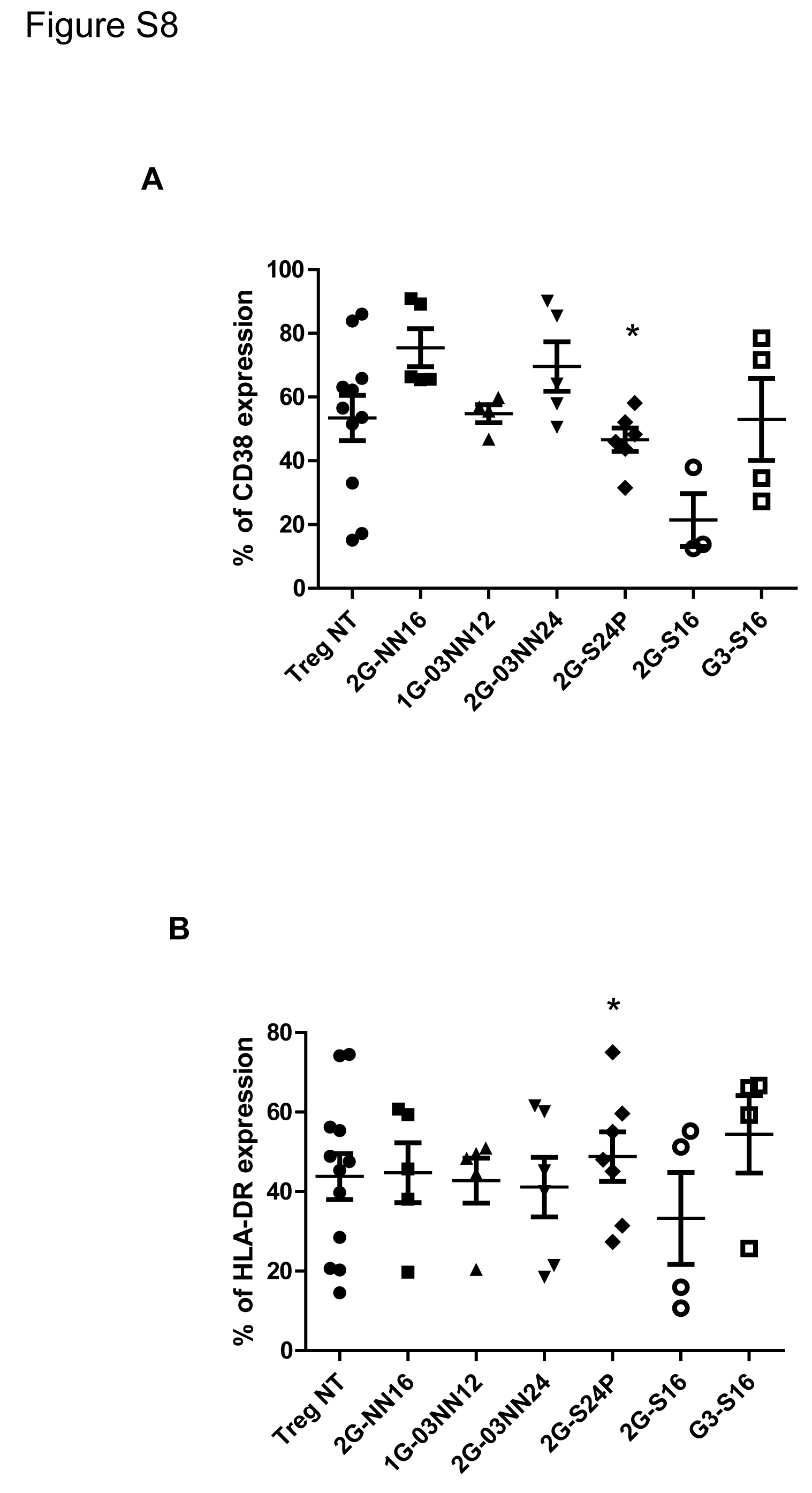

Supplement: S8 Fig — (TIF) [file pone.0145760.s008.tif]
